# Supplementary material for: Calcium and bicarbonate signaling pathways have pivotal, resonating roles in matching ATP production to demand
Source: eLife. 2023 Jun 5;12:e84204. doi: 10.7554/eLife.84204 (PMC10284600; doi:10.7554/eLife.84204)

Figure 3C_ RAP1_ SourceData2

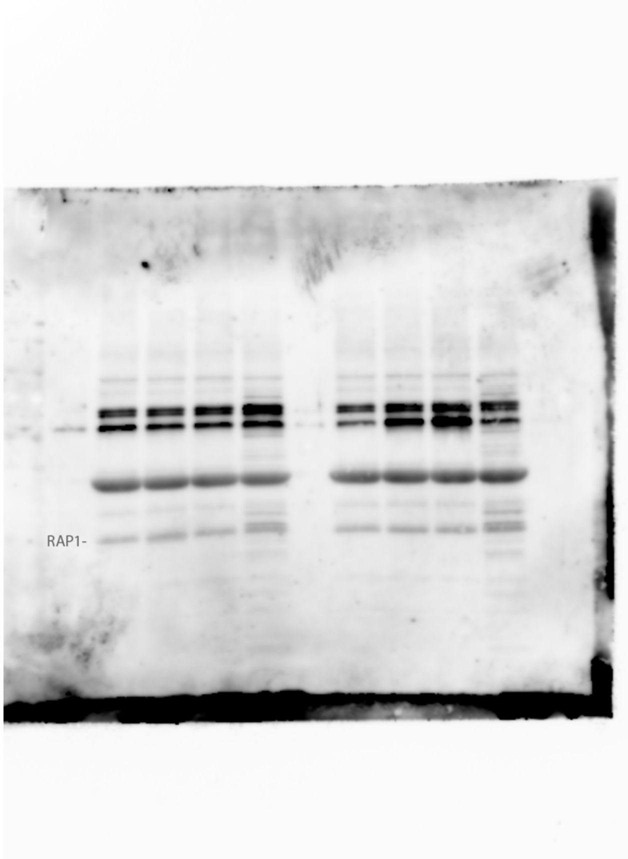

Figure 3C_ RAP1_SourceData3

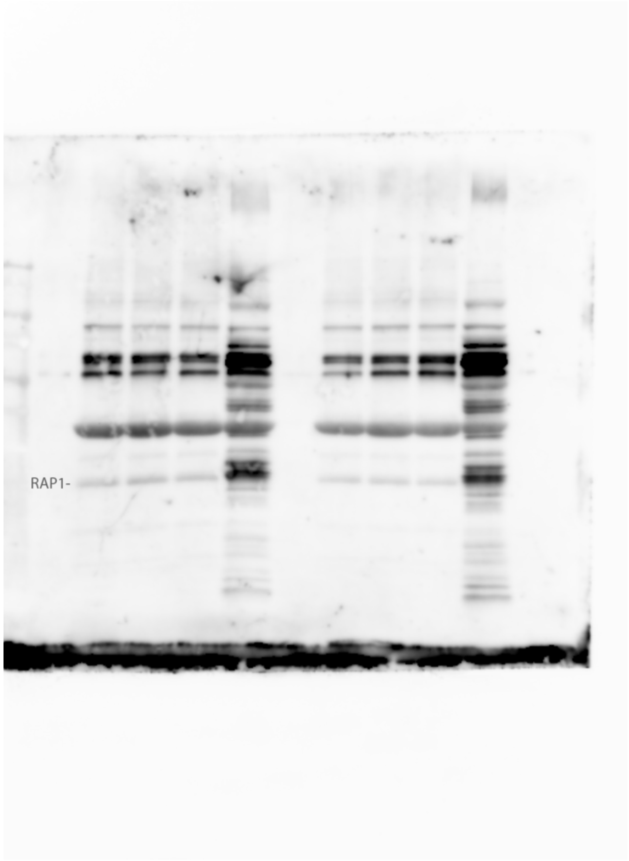

Figure 3C_RAP1_ SourceData4

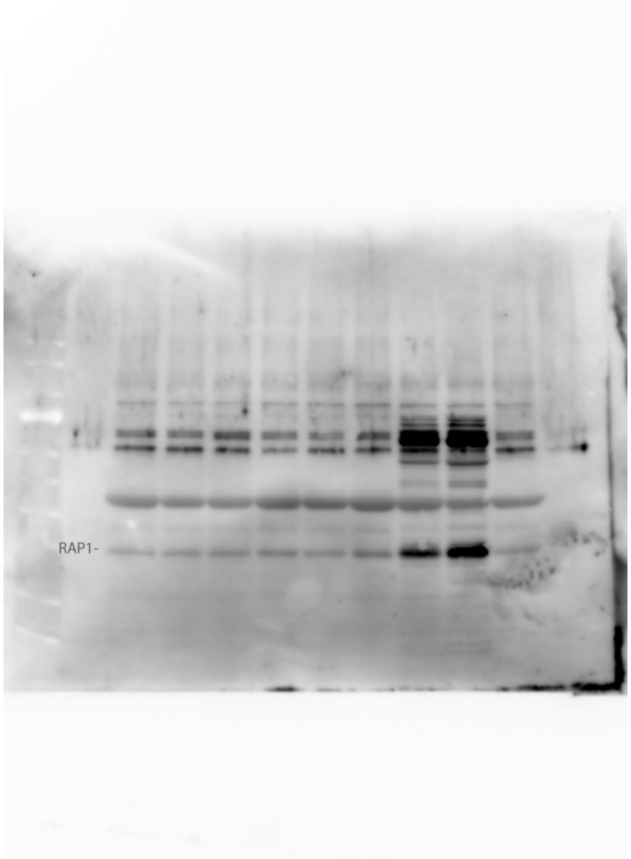

Figure 3C_ RAP1_ SourceData5

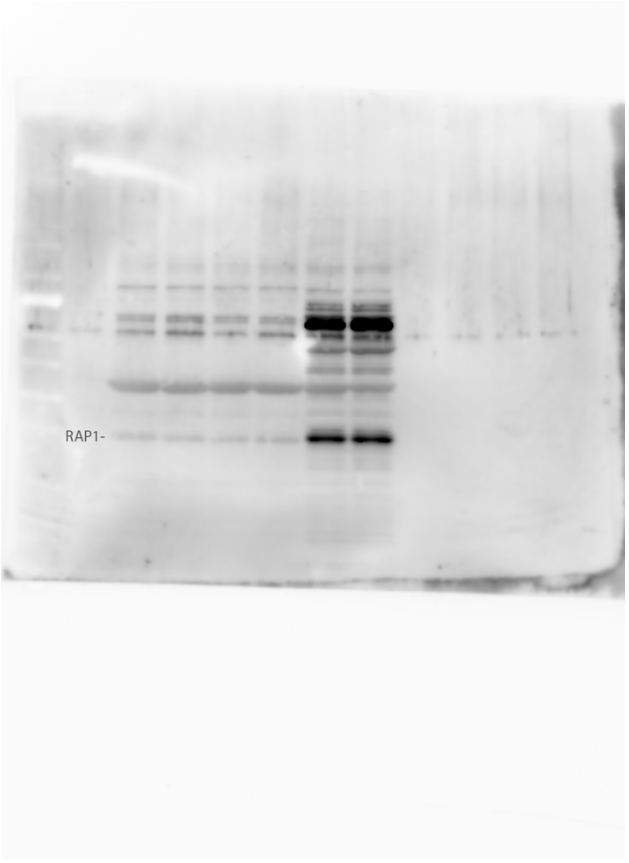

Supplement: Figure 3—source data 1. [file elife-84204-fig3-data1.zip › Figure_3C_RAP1_SourceData1.pdf]
