# Supplementary figures and images for: Calcium and bicarbonate signaling pathways have pivotal, resonating roles in matching ATP production to demand
Source: eLife. 2023 Jun 5;12:e84204. doi: 10.7554/eLife.84204 (PMC10284600; doi:10.7554/eLife.84204)

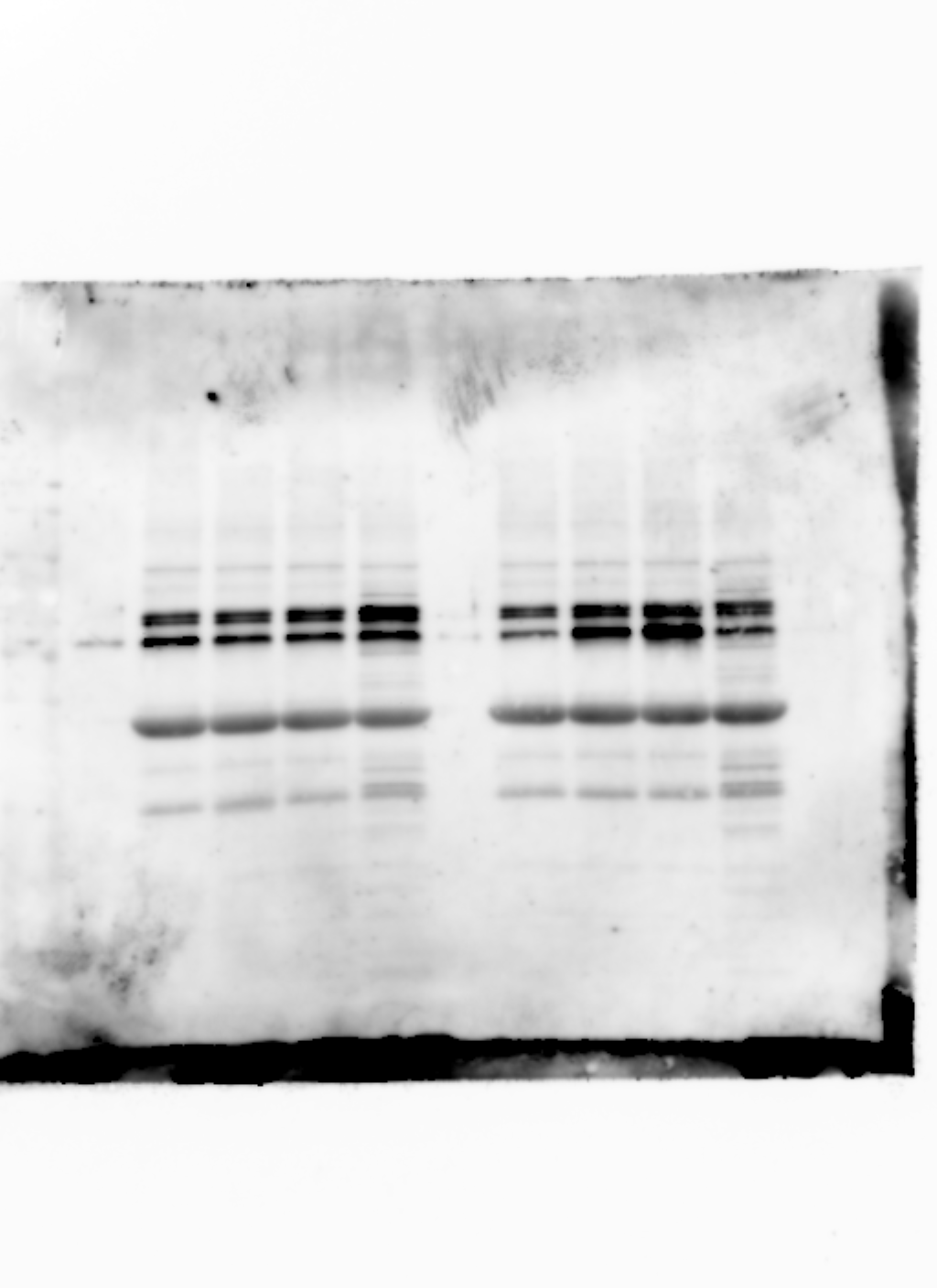

Supplement: Figure 3—source data 2. [file elife-84204-fig3-data2.zip › Figure_3C_RAP1_1_SourceData2.tif]

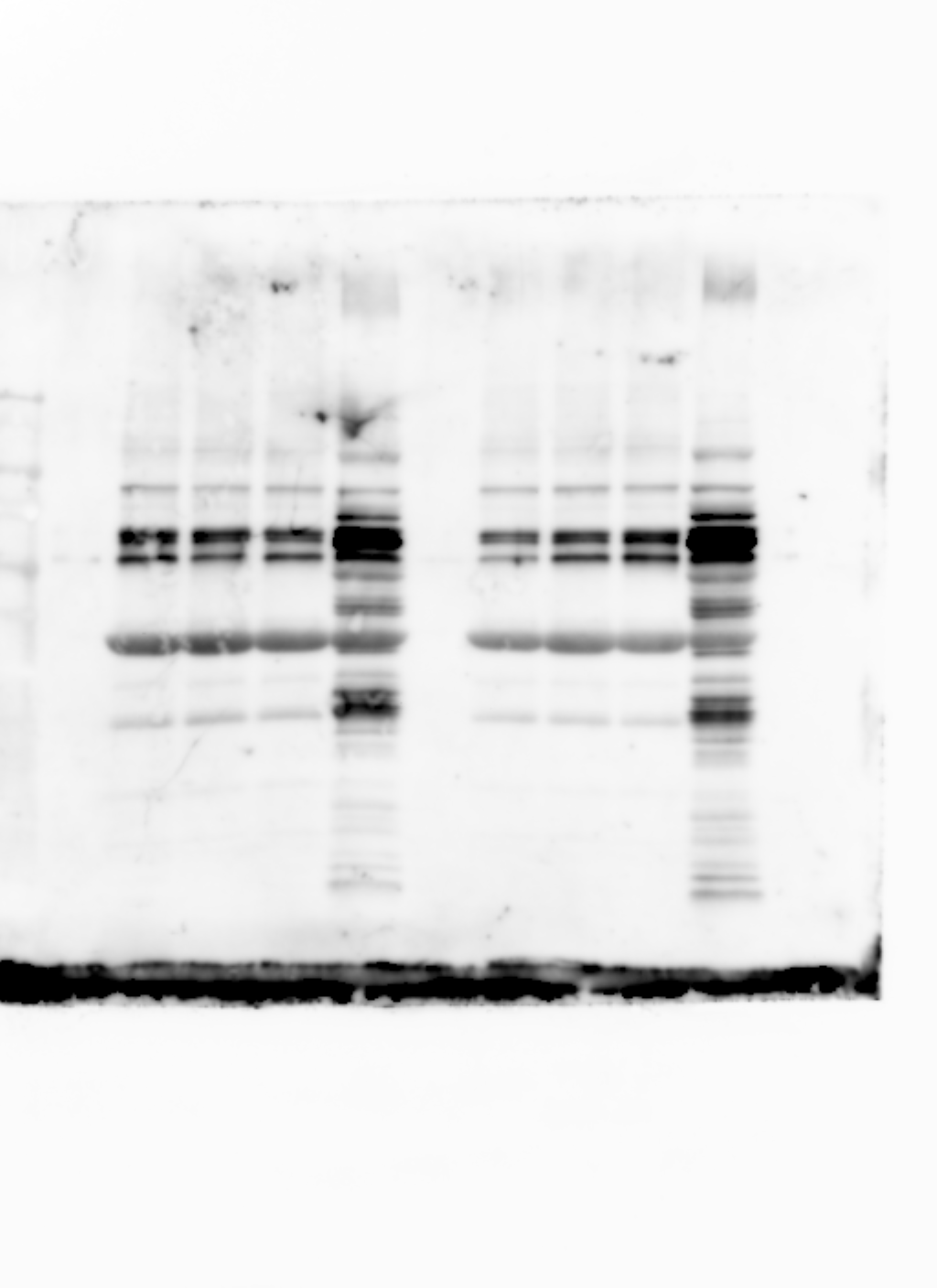

Supplement: Figure 3—source data 3. [file elife-84204-fig3-data3.zip › Figure_3C_RAP1_2_SourceData3.tif]

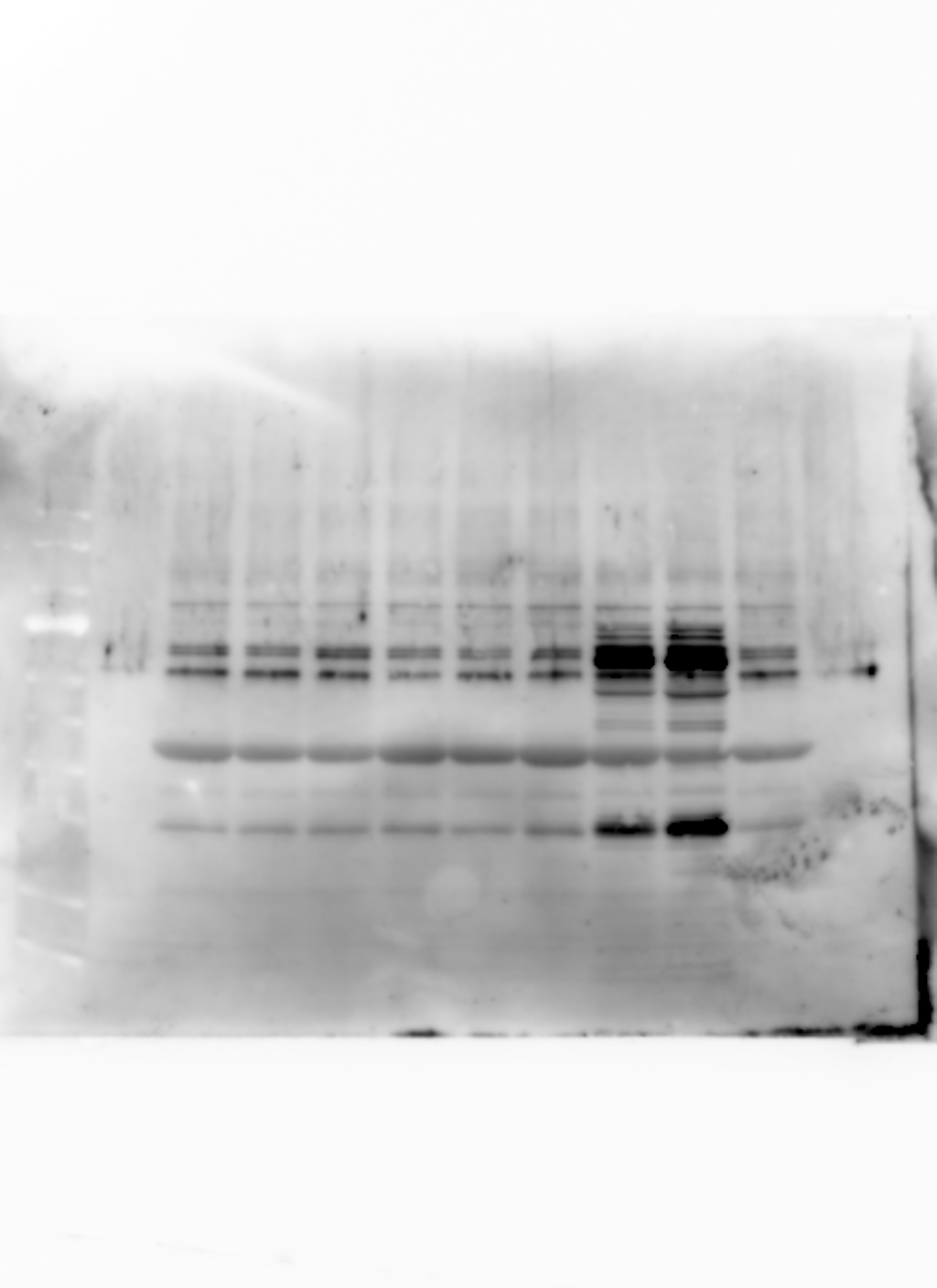

Supplement: Figure 3—source data 4. [file elife-84204-fig3-data4.zip › Figure_3C_RAP1_3_SourceData4.tif]

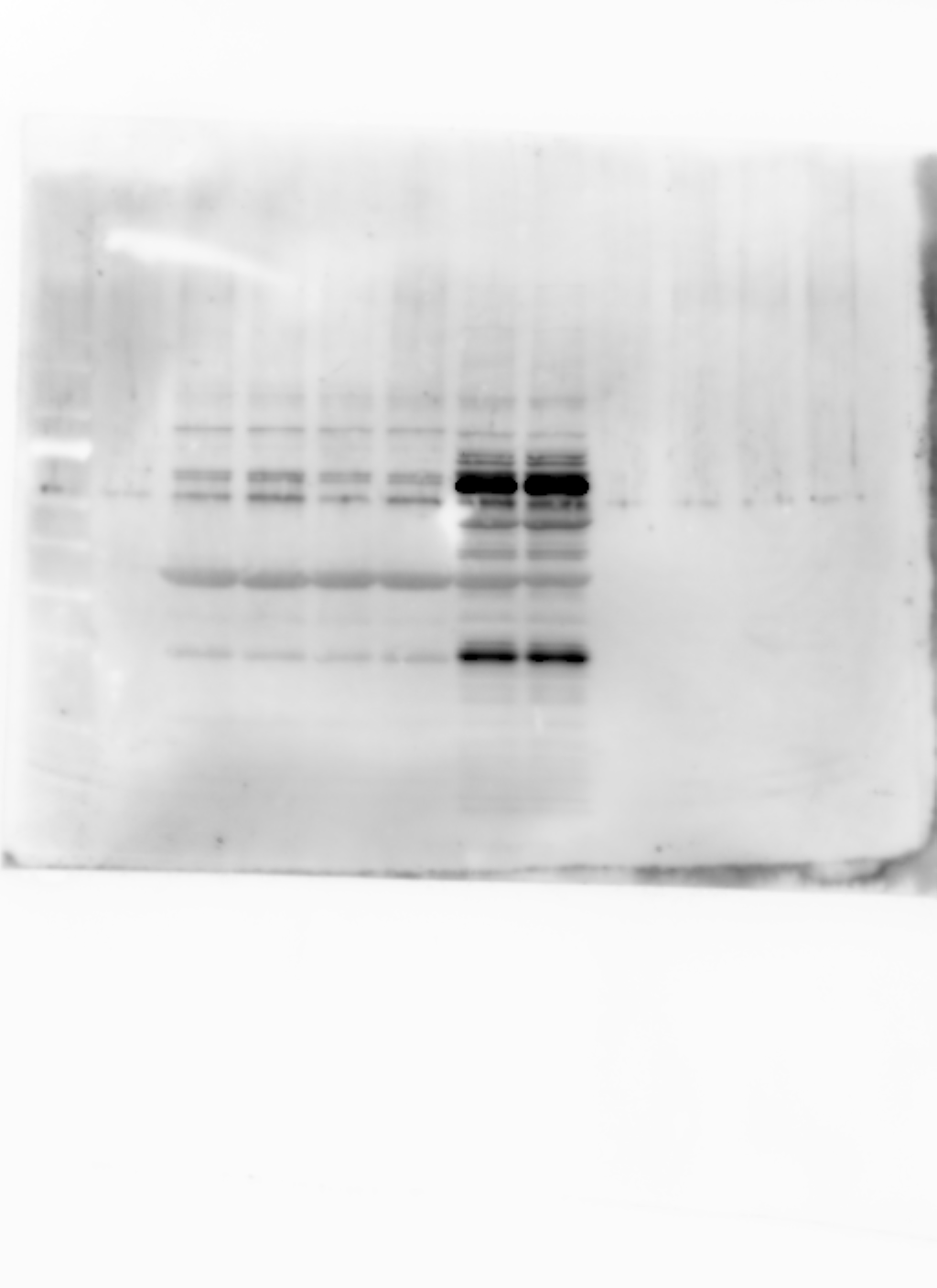

Supplement: Figure 3—source data 5. [file elife-84204-fig3-data5.zip › Figure_3C_RAP1_4_SourceData5.tif]

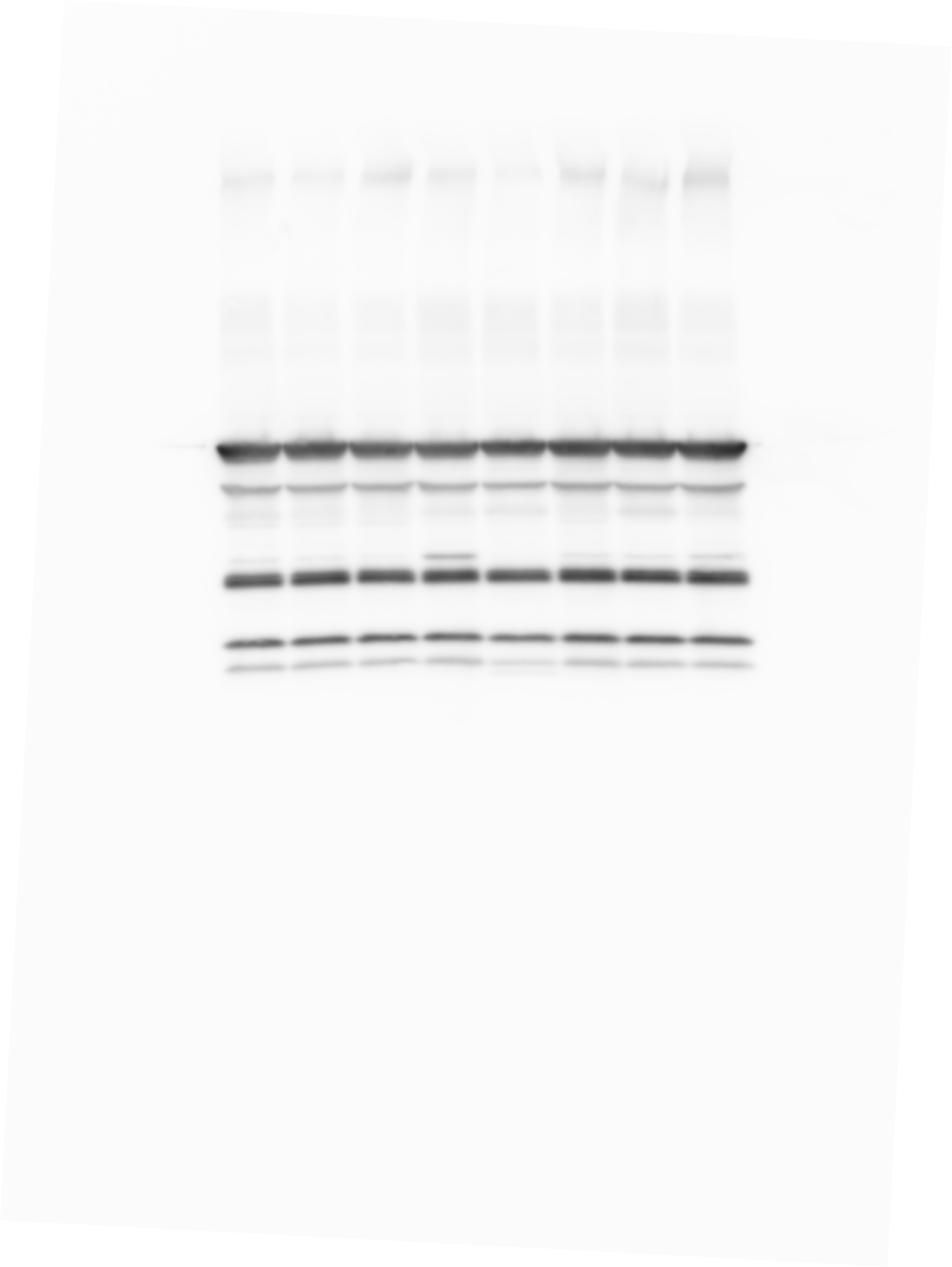

Supplement: Figure 6—figure supplement 2—source data 2. [file elife-84204-fig6-figsupp2-data2.zip › Figure_S3A_Posterior_wall_ETC_SourceData2.tif]

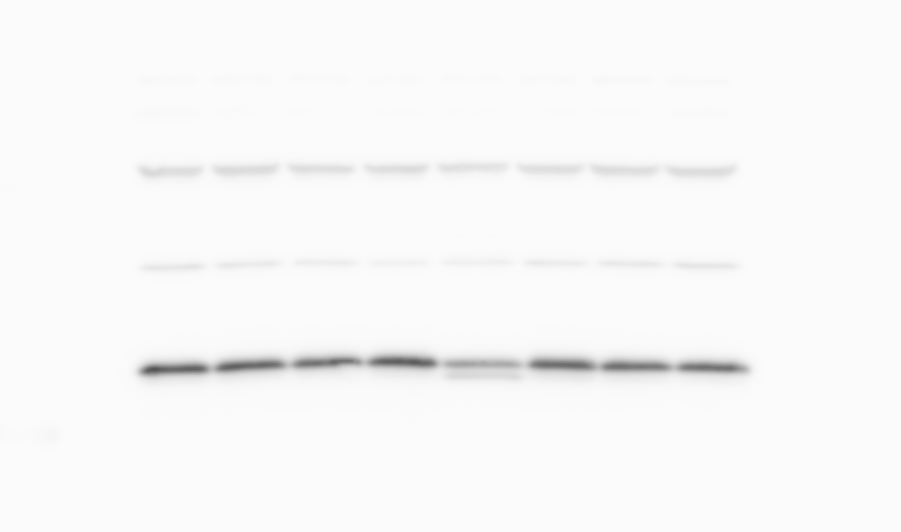

Supplement: Figure 6—figure supplement 2—source data 3. [file elife-84204-fig6-figsupp2-data3.zip › Figure_S3A_Posterior_wall_Tom20_SourceData3.tif]

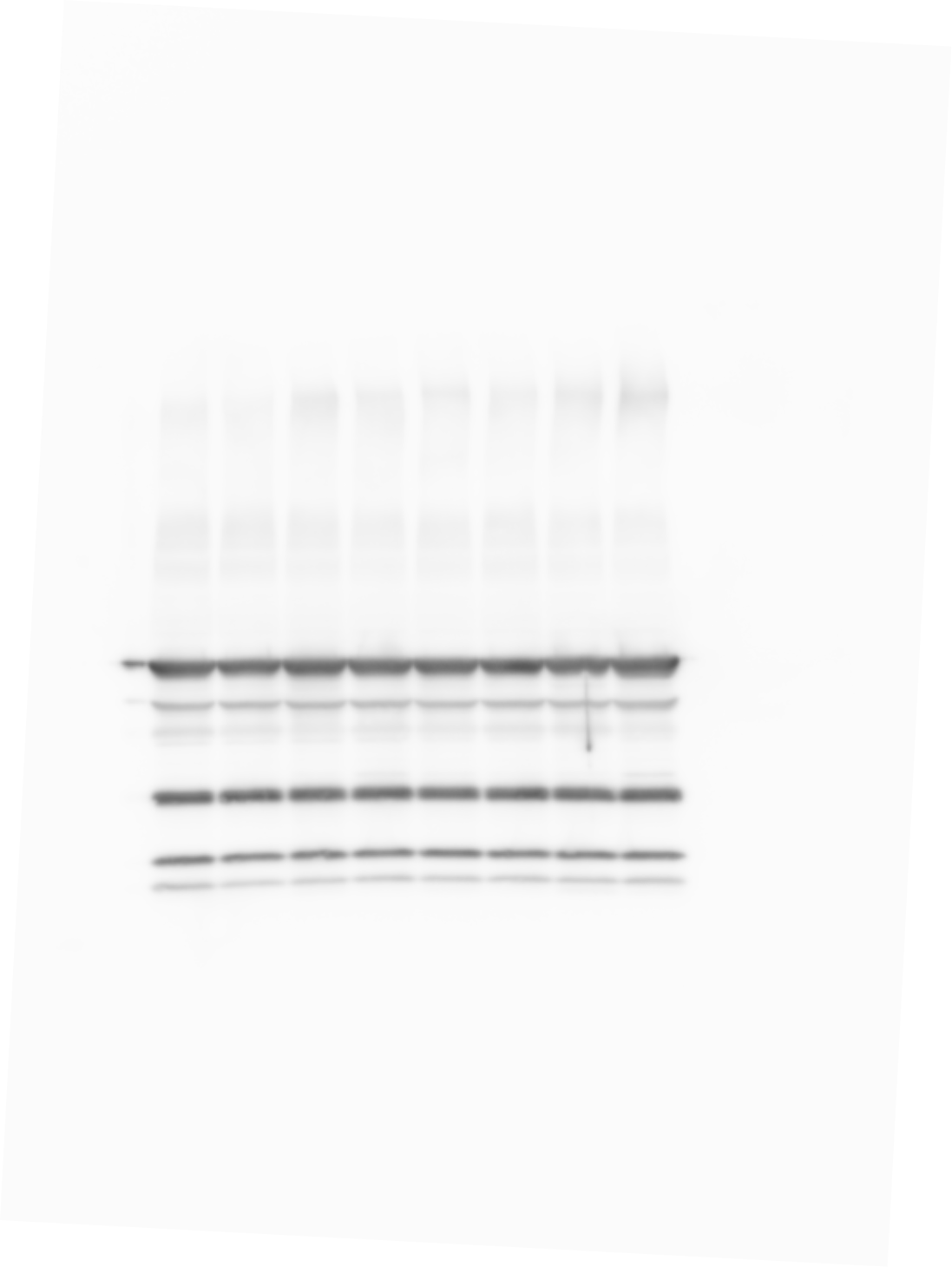

Supplement: Figure 6—figure supplement 2—source data 4. [file elife-84204-fig6-figsupp2-data4.zip › Figure_S3A_Septal_wall_ETC_SourceData4.tif]

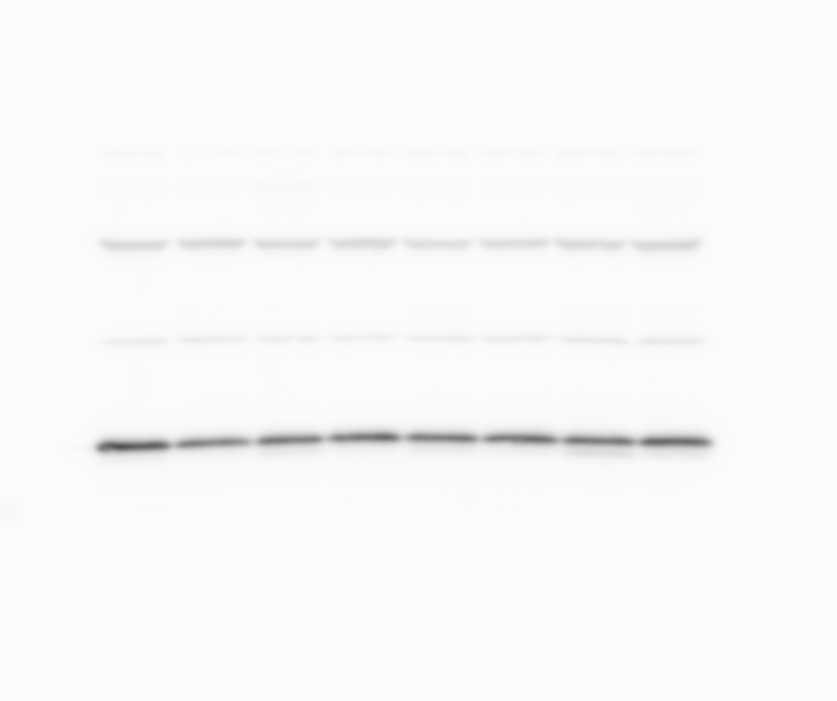

Supplement: Figure 6—figure supplement 2—source data 5. [file elife-84204-fig6-figsupp2-data5.zip › Figure_S3A_Septal_wall_Tom20_SourceData5.tif]
