# Supplementary material for: Calcium and bicarbonate signaling pathways have pivotal, resonating roles in matching ATP production to demand
Source: eLife. 2023 Jun 5;12:e84204. doi: 10.7554/eLife.84204 (PMC10284600; doi:10.7554/eLife.84204)

Figure S3A - posterior wall - ETC (SourceData2)

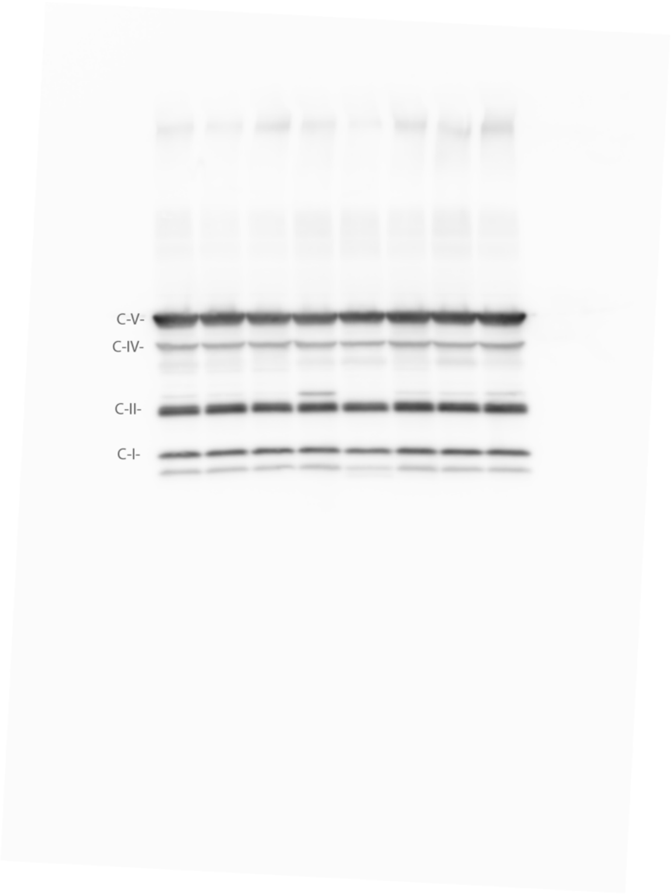

Figure S3A - posterior wall - Tom20 (SourceData3)

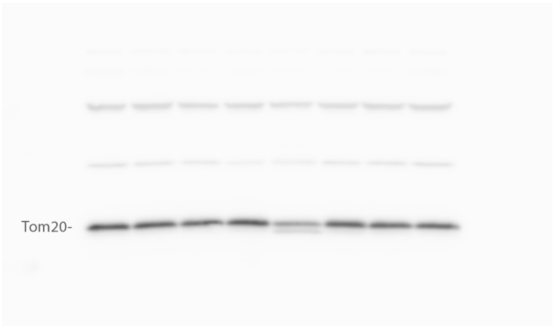

Figure S3A - Septal wall - ETC (SourceData4)

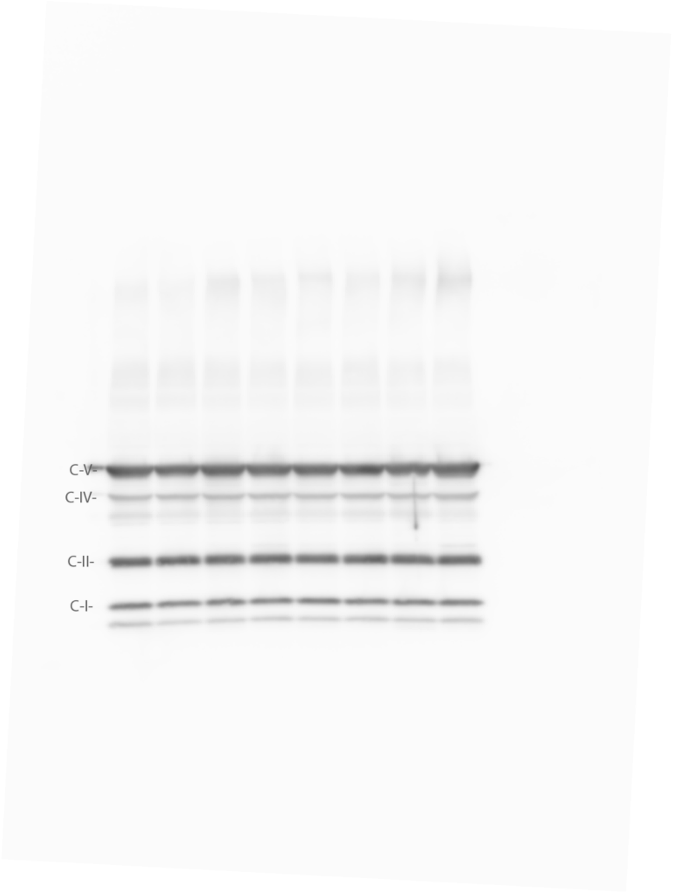

Figure S3A - Septal wall - Tom20 (SourceData5)

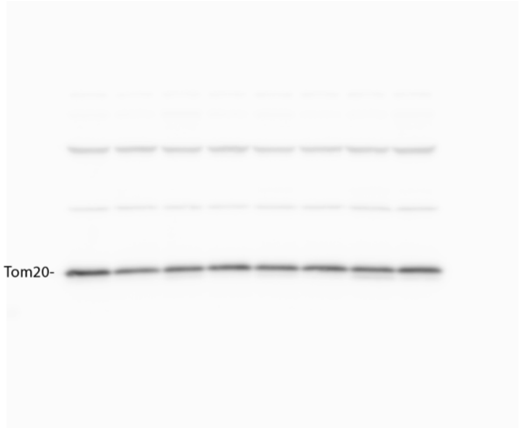

Supplement: Figure 6—figure supplement 2—source data 1. [file elife-84204-fig6-figsupp2-data1.zip › Figure_S3ASourceData1.pdf]
