# Supplementary material for: Calcium and bicarbonate signaling pathways have pivotal, resonating roles in matching ATP production to demand
Source: eLife. 2023 Jun 5;12:e84204. doi: 10.7554/eLife.84204 (PMC10284600; doi:10.7554/eLife.84204)
Supplement: Supplementary file 1. [file elife-84204-supp1.docx]

Supplementary File 1

| Citation | Native Mitochondrial sAC localization | Role of PKA | Physiological role of sAC signaling |
| --- | --- | --- | --- |
| Manfredi and coworkers^34, 40-42^ | Matrix | PKA-dependent | Sensing nutrient availability |
| Balaban and coworkers^43^ | ND | PKA-independent | ND |
| Lefkimmiatis  ^23, 44^ | ND | PKA-independent | ND |
| Brenner and coworkers ^45^ | ND | PKA-independent | Regulates calcium accumulation, permeability transition and cell death |
| This paper | mitochondrial inter membrane space | PKA-independent | Sensing substrate consumption - mechano-metabolic sensor |

**Supplementary File 1.** Mechanistic findings by investigations of sAC and its role in mitochondria.
